# Perspectives on High-Value Care Education Among US Medical Students

**DOI:** 10.1001/jamanetworkopen.2025.39462

**Published:** 2025-10-27

**Authors:** Hannah K. Bassett, Michelle Chen, Shivani Jain, Anitra Karthic, Karthik Ramesh, Hannah Shenton, September Wallingford, Allison Heacock

**Affiliations:** 1 Division of Hospital Medicine, Department of Pediatrics, Stanford University School of Medicine, Palo Alto, California; 2 Now with Department of Medicine, University of California, Los Angeles School of Medicine, Los Angeles; 3 University of California, San Diego School of Medicine, San Diego; 4 Louisiana State University Health Sciences Center-New Orleans School of Medicine, New Orleans; 5 The Ohio State University College of Medicine, Columbus; 6 Now with Department of Family Medicine, The University of Arizona College of Medicine-Phoenix, Phoenix; 7 Costs of Care, Cambridge, Massachusetts; 8 Division of Hospital Medicine, Department of Internal Medicine and Section of Hospital Medicine, Department of Pediatrics, The Ohio State University College of Medicine, Columbus

## Abstract

This survey study examines the perceived prevalence and effectiveness of formal and informal high-value care curricula in core clerkships across US medical schools.

## Introduction

Training clinicians to be competent in high-value care (HVC) is critical as US health care costs grow unsustainably, and low-value practices and overspending remain pervasive.^1,2^ Because the early medical educational environment has a lasting influence on clinician practice patterns,^3^ integrating HVC curricula into the clinical years of undergraduate medical education (UME) may be an optimal time to pair value-based reasoning with developing clinical skills and ensure all students receive a foundation in value-based care. We assessed the perceived prevalence and effectiveness of formal and informal HVC curricula in core clerkships across US medical schools.

## Methods

This cross-sectional survey study of third-year and fourth-year medical students was conducted from January to February 2024. Students were eligible if they participated in the national Students and Trainees Advocating for Resource Stewardship (STARS) program during their preclinical years. The survey was adapted from the Clerkship Directors in Internal Medicine annual survey.^4^ Cognitive interviews and pilot testing by eligible students led to minor revisions. The final survey (eAppendix in Supplement 1) included 21 questions (not all data reported here). Sociodemographic information was not collected as these data were not thought to be relevant to data interpretation. The survey was hosted on Qualtrics version 2024 and was distributed electronically. Responses were anonymous, and participants received a $20 gift card; participants provided consent by responding to the survey. This study was deemed exempt by the institutional review board at the University of Texas at Austin Dell Medical School. This study followed the American Association for Public Opinion Research (AAPOR) reporting guideline.

Partially completed surveys at the end of the response period were included in the analysis (American Association for Public Opinion Research Cooperation Rate 2).^5^ Because this was an exploratory study and our eligible population was finite, we did not calculate an a priori sample size. Descriptive statistics summarized responses. Reported percentages are based on the number of respondents who answered each question.

## Results

Of 229 eligible students, 119 (52%) responded to the survey, representing 40 US medical schools. Regarding their core clerkships, 56 of 119 students (47%) reported at least 1 had a formal HVC curriculum, 105 (88%) reported at least 1 had an informal HVC curriculum, and 11 (9%) reported no exposure to any HVC curriculum. The prevalence of HVC curricula varied widely across clerkships (Table). Internal medicine and family or community medicine clerkships were perceived as most effectively incorporating HVC teaching, whereas emergency medicine and general surgery clerkships had the greatest discrepancy between the perceived need for effective HVC education and its current implementation (Figure). Overall, 63% of respondents (73 of 116 students) reported being extremely or somewhat dissatisfied with their HVC education during core clerkships.

**Table.** Proportion of Completed Core Clerkships and Perceived Prevalence of HVC Curricula

Core clerkship^a^	Respondents, No. (%)		
	Completed clerkship (N = 119)	Reported prevalence of formal HVC curriculum^b^^,^^c^	Reported prevalence of informal HVC curriculum^c^^,^^d^
Internal medicine	108 (91)	40 (37)	82 (76)
Pediatrics	105 (88)	10 (10)	42 (40)
Psychiatry	101 (85)	3 (3)	11 (11)
General surgery	99 (83)	8 (8)	30 (30)
Obstetrics and gynecology	99 (83)	6 (6)	19 (19)
Family and/or community medicine	90 (76)	34 (38)	55 (61)
Neurology	85 (71)	4 (5)	18 (21)
Emergency medicine	48 (40)	8 (17)	18 (38)

Abbreviation: HVC, high-value care.

^a^ Respondents were asked to indicate only those clerkships that were considered core clerkships at their medical school.

^b^ Formal HVC curriculum defined as teaching that was planned or required as part of clerkship expectations (eg, didactic sessions or online modules).

^c^ Percentages are based on the number of respondents who completed that clerkship.

^d^ Informal HVC curriculum defined as teaching that was not planned or required as part of clerkship expectations (eg, discussions on rounds or teaching pearls).

**Figure.**
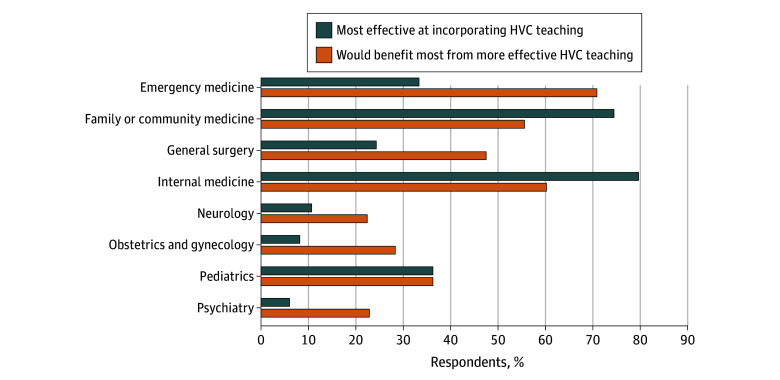
Respondents’ Perceptions of Effective High-Value Care (HVC) Teaching Within Core Clerkships Respondents were asked to choose up to 3 clerkships for each question. The denominator for each clerkship is the number of respondents who had completed that clerkship at the time of responding to the survey (Table).

## Discussion

More than one-half of the clerkship-level medical students in this survey study reported their core clerkships had no formal HVC curriculum. Although almost 90% reported experiencing informal HVC education, having students rely primarily on ad hoc teaching introduces variability in experiences and knowledge. The consistency and clear objectives of a formal curriculum are needed to provide a foundation of clerkship-specific HVC knowledge that students can build upon through clinical experiences. Although our findings support the need for universal improvement in HVC education during the clinical years of UME, they also highlight specialty-specific opportunities, as certain clerkships were notable outliers in the perceived prevalence and need for a HVC curriculum.

Limitations of this study include a response rate of 52%, raising the possibility of response bias, and the retrospective nature of the survey, raising the possibility of recall bias. Additionally, this study is limited to the experiences and reflections of clinical year medical students and, therefore, may not comprehensively represent curricular content in their core clerkships. However, student perspectives are essential when comprehensively evaluating curricula and have largely been missing from the literature on this topic. Our findings highlight the need for a structured and consistent strategy for integrating HVC education within clinical UME to cultivate physicians who are equipped to deliver high-value, cost-conscious care.

## Notes

Supplement 1. eAppendix. Survey Instrument

Supplement 2. Data Sharing Statement
