# Supplementary material for: Perspectives on High-Value Care Education Among US Medical Students
Source: JAMA Netw Open. 2025 Oct 27;8(10):e2539462. doi: 10.1001/jamanetworkopen.2025.39462 (PMC12559963; doi:10.1001/jamanetworkopen.2025.39462)
Supplement: Supplement 1. — eAppendix. Survey Instrument [file jamanetwopen-e2539462-s001.pdf]

Supplemental Online Content

Bassett HK, Chen M, Jain S, et al. Perspectives on high-value care education among US medical students. *JAMA Netw Open*. 2025;8(10):e2539462.
doi:10.1001/jamanetworkopen.2025.39462

eAppendix. Survey Instrument

This supplemental material has been provided by the authors to give readers additional information about their work.

© 2025 Bassett HK et al. *JAMA Network Open*.

eAppendix. Survey Instrument

For the purposes of this survey, "**high-value care (HVC)**" is defined as the following:

- understanding and choosing clinical interventions that maximize benefits, minimize harms, and reduce costs
- incorporating patient values and addressing their concerns as part of clinical decision making
- identifying system level opportunities to improve clinical outcomes and reduce health care waste

When answering questions, please reflect on your experiences only during your **core clerkships**.

1. Which medical school do you attend?
   Free text

2. Which of the following **core clerkships** have you completed? *Check all that apply. Note: We recognize not all of these may be considered core clerkships at your institution. Please only choose those that are applicable to you.*
   a. Emergency Medicine
   b. Family Medicine / Community Medicine
   c. General Surgery
   d. Internal Medicine
   e. Neurology
   f. Obstetrics / Gynecology
   g. Pediatrics
   h. Psychiatry
   i. Other: free text

3. At least one core clerkship had **formal educational components** focused on HVC topics (i.e. teaching that was planned or required as part of clerkship expectations, e.g didactic sessions, online modules).
   a. Yes (next question)
   b. No (skip logic to question 9)

4. Which core clerkships had **formal educational components** focused on HVC concepts? *Check all that apply.*
   a. Internal Medicine
   b. Pediatrics
   c. Surgery
   d. Psychiatry
   e. Neurology
   f. OB/Gyn
   g. Family Medicine / Community Medicine
   h. Emergency Medicine

5. Which of the following methods were used in your core clerkships to **formally teach** HVC concepts? *Check all that apply*.
   a. Structured didactic sessions (e.g. lecture, problem based learning, journal club)
   b. Assigned reading materials
   c. Web-based (online) modules
   d. Simulation-based education (e.g. case scenarios with standardized or simulated patients)
   e. Point of care decision making tools or standardized discussion in the clinical setting (e.g. badge cards, rounding/visit checklist in EHR)

© 2025 Bassett HK et al. *JAMA Network Open*.

   f. Targeted review of patient charts, billing, or orders to highlight HVC (e.g. M&M)
   g. Structured reflection on clinical experience with subsequent discussion or feedback
   h. Other (free text)

6. How effective were each of the following methods in **teaching HVC**? *For each method chosen in question 5, respondents answered on a Likert scale from not effective at all to extremely effective.*
   a. Structured didactic sessions (e.g. lecture, problem based learning, journal club)
   b. Assigned reading materials
   c. Web-based (online) modules
   d. Point of care decision making tools or standardized discussion on rounds (e.g. badge cards, rounding checklist in EHR)
   e. Simulation-based education (e.g. case scenarios with standardized or simulated patients)
   f. Targeted review of patient chart, billing, or orders to highlight HVC (e.g. M&M)
   g. Structured reflection on clinical experience with subsequent discussion or feedback
   h. Other (free text)

7. How frequently did these **formal educational components** occur on each rotation? *Answered for each rotation respondents indicated had formal educational components in question 4.*
   a. Daily
   b. 2-3 times per week
   c. Once per week
   d. 2-3 times per rotation
   e. Once per rotation

8. At least one of my core clerkships had **informal educational components** focused on HVC concepts (i.e. teaching that was not planned or required as part of clerkship expectations, e.g. discussions on rounds or in clinic, teaching pearls, chalk talks, etc).
   a. Yes
   b. No (skip logic to question 12)

9. Which core clerkships had **informal educational components** focused on HVC concepts? *Check all that apply.*
   a. Emergency Medicine
   b. Family Medicine / Community Medicine
   c. General Surgery
   d. Internal Medicine
   e. Neurology
   f. Obstetrics / Gynecology
   g. Pediatrics
   h. Psychiatry
   i. Other (free text)

10. How frequently did these **informal educational components** occur on each rotation? *Answered for each rotation respondents indicated had informal educational components in question 9.*
    a. Daily
    b. 2-3 times per week
    c. Once per week
    d. 2-3 times per rotation
    e. Once per rotation

11. Based on your experience, who is **primarily teaching or facilitating learning** about HVC on your core clerkships?
    a. Attendings / faculty

© 2025 Bassett HK et al. *JAMA Network Open*.

b. Fellows
c. Residents
d. Peers / medical students
e. Other staff (e.g. case managers, social workers)

12. Which **HVC topics** were taught (either formally or informally) during your core clerkships? *Check all that apply.*
    a. Defining value
    b. Healthcare costs and payment models
    c. Balancing benefits with harms and costs
    d. Medications and value (e.g. generic vs brand name meds, cost, adherence)
    e. Barriers to high value care
    f. Cost-effectiveness analysis
    g. Preventive care and value
    h. Overuse and misuse of diagnostic tests (e.g. labs, imaging)
    i. Health system-level factors that impact value for patients
    j. Other (free text)

13. Of the core clerkships you have completed, which ones **most effectively incorporated** HVC teaching and learning? *Please rank up to 3.*
    a. Emergency Medicine
    b. Family Medicine / Community Medicine
    c. General Surgery
    d. Internal Medicine
    e. Neurology
    f. Obstetrics / Gynecology
    g. Pediatrics
    h. Psychiatry
    i. Other (free text)

14. Of the core clerkships you have completed, which ones would **benefit the most** from more effective teaching on HVC topics? *Please rank up to 3.*
    a. Emergency Medicine
    b. Family Medicine / Community Medicine
    c. General Surgery
    d. Internal Medicine
    e. Neurology
    f. Obstetrics / Gynecology
    g. Pediatrics
    h. Psychiatry
    i. Other (free text)

15. What methods were used to **assess your competency** in HVC during your core clerkships? *Check all that apply.*
    a. Knowledge test (i.e. multiple choice exam)
    b. Interactive computer-based / online modules
    c. Standardized patient encounters (e.g. OSCE)
    d. Component of clinical clerkship evaluation (either formative or summative)
    e. Other (free text)_
    f. My competency in HVC was not assessed

16. How effective were each of the following assessment methods in providing feedback on your competency in HVC? *For each method chosen in question 15, respondents answered on a Likert scale from not effective at all to extremely effective.*

© 2025 Bassett HK et al. *JAMA Network Open*.

a. Knowledge test (e.g. multiple choice exam)
b. Interactive computer-based / online modules
c. Standardized patient encounters (e.g. OSCE)
d. Component of clinical clerkship evaluation (either formative or summative)
e. Other (free text)

17. How satisfied are you **overall** with the HVC curriculum in your core clerkships?
    a. Extremely satisfied
    b. Somewhat satisfied
    c. Neither satisfied nor dissatisfied
    d. Somewhat dissatisfied
    e. Extremely dissatisfied

18. Which of the following HVC topics do you think are the **most important to learn** in the context of your core clerkships (i.e. how these topics relate to each specialty)? *Please rank your top 3.*
    a. Defining value
    b. Healthcare costs and payment models
    c. Balancing benefits with harms and costs
    d. Medications and value (e.g. generic vs brand name meds, cost, adherence)
    e. Barriers to high value care
    f. Cost-effectiveness analysis
    g. Preventive care and value
    h. Overuse and misuse of diagnostic tests (e.g. labs, imaging)
    i. Health system-level factors that impact value for patients
    j. Other (free text)

19. Which of the following methods do you think would be the **most effective in teaching** HVC concepts during your core clerkships? *Please rank your top 3.*
    a. Structured didactic sessions (e.g. lecture, problem based learning, journal club)
    b. Targeted review of patient chart, billing, or orders to highlight HVC (e.g. M&M)
    c. Assigned reading materials
    d. Web-based (online) modules
    e. Point of care decision making tools or standardized discussion on rounds (e.g. badge cards, rounding/visit checklist in EHR)
    f. Simulation-based education (e.g. case scenarios with standardized or simulated patients)
    g. Structured reflection on clinical experience with subsequent discussion or feedback
    h. Other (free text)

20. What do you think are the **main barriers** in teaching and/or learning HVC during core clerkships? *Please rank your top 3.*
    a. Lack of attending / faculty expertise or interest
    b. Lack of attending / faculty bandwidth
    c. Lack of fellow / resident expertise or interest
    d. Lack of fellow / resident bandwidth
    e. Lack of adequate HVC resources
    f. Clinical demands of core clerkships
    g. Curricular demands of core clerkships
    h. Other (free text)

21. Please provide any additional comments about high value care education in your core clerkships.
    Free text box.

© 2025 Bassett HK et al. *JAMA Network Open*.
