# Supplementary material for: Perspectives on High-Value Care Education Among US Medical Students
Source: JAMA Netw Open. 2025 Oct 27;8(10):e2539462. doi: 10.1001/jamanetworkopen.2025.39462 (PMC12559963; doi:10.1001/jamanetworkopen.2025.39462)
Supplement: Supplement 2. — Data Sharing Statement [file jamanetwopen-e2539462-s002.pdf]

Data Sharing Statement

Bassett. Perspectives on High-Value Care Education Among US Medical Students. *JAMA Netw Open*. Published October 27, 2025. doi:10.1001/jamanetworkopen.2025.39462

Data
Data available: No
